# Assessment of prostate cancer prognostic Gleason grade group using zonal‐specific features extracted from biparametric MRI using a KNN classifier

**DOI:** 10.1002/acm2.12542

**Published:** 2019-02-03

**Authors:** Carina Jensen, Jesper Carl, Lars Boesen, Niels Christian Langkilde, Lasse Riis Østergaard

**Affiliations:** ^1^ Department of Medical Physics, Oncology Aalborg University Hospital Aalborg Denmark; ^2^ Department of Oncology Naestved Sygehus Zealand University Hospital Roskilde Denmark; ^3^ Department of Urology Herlev Gentofte University Hospital Herlev Denmark; ^4^ Department of Urology Aalborg University Hospital Aalborg Denmark; ^5^ Department of Health Science and Technology Aalborg University Aalborg Denmark

**Keywords:** Gleason grade, Gleason Score, KNN, MRI, prostate cancer

## Abstract

**Purpose:**

To automatically assess the aggressiveness of prostate cancer (PCa) lesions using zonal‐specific image features extracted from diffusion weighted imaging (DWI) and T2W MRI.

**Methods:**

Region of interest was extracted from DWI (peripheral zone) and T2W MRI (transitional zone and anterior fibromuscular stroma) around the center of 112 PCa lesions from 99 patients. Image histogram and texture features, 38 in total, were used together with a k‐nearest neighbor classifier to classify lesions into their respective prognostic Grade Group (GG) (proposed by the International Society of Urological Pathology 2014 consensus conference). A semi‐exhaustive feature search was performed (1–6 features in each feature set) and validated using threefold stratified cross validation in a one‐versus‐rest classification setup.

**Results:**

Classifying PCa lesions into GGs resulted in AUC of 0.87, 0.88, 0.96, 0.98, and 0.91 for GG1, GG2, GG1 + 2, GG3, and GG4 + 5 for the peripheral zone, respectively. The results for transitional zone and anterior fibromuscular stroma were AUC of 0.85, 0.89, 0.83, 0.94, and 0.86 for GG1, GG2, GG1 + 2, GG3, and GG4 + 5, respectively.

**CONCLUSION:**

This study showed promising results with reasonable AUC values for classification of all GG indicating that zonal‐specific imaging features from DWI and T2W MRI can be used to differentiate between PCa lesions of various aggressiveness.

## 1 INTRODUCTION

Prostate cancer (PCa) remains the most common noncutaneous cancer among men and one of the most common causes of cancer‐related deaths.^1^ PCa ranges from nonsignificant indolent to an aggressive cancer with fatal outcome.^2^ The histopathological aggressiveness of PCa is graded by the Gleason Score (GS), which is a powerful predictor of progression, mortality, and outcomes of the disease.^3^ The GS describes the degree of differentiation and growth patterns of cells in the tumor.^2^ Higher GS indicates higher level of aggression with worse prognosis.^2^ The GS from prostate biopsies is used for clinical decision‐making, treatment selection, and prediction of outcomes for patients. However, due to the random sampling when obtaining prostate biopsies, the GS differs from that determined after radical prostatectomy (RP).^3^, ^4^ At the time of diagnosis, the ability to distinguish between indolent, intermediate, and aggressive PCa is limited, leading to incorrect risk stratification and possible over‐ and undertreatment.^5^

Radical treatment approaches, such as RP or radiation therapy, are common treatment options for PCa patients.^4^ However, due to the adverse side effects of radical treatments, such as urinary incontinence, bowel problems, and erectile dysfunction, more conservative treatments, such as active surveillance (AS), are increasingly being considered for men with relatively indolent cancers.^3^

Of patients initially enrolled in AS, up to 33% are initially understaged or has disease progression within 2–5 years leading to active treatment. Furthermore, significant cancers are found in RP specimens in 73% of patients who are initially eligible for AS.^4^ Primary focal therapy like focal brachytherapy or cryotherapy, is increasingly considered as an alternative treatment option with less morbidity while still achieving cancer control for selected patients with low and intermediate‐risk PCa.^4^ Thus, accurate pretherapeutic risk‐assessment is crucial for correct patient‐tailored treatment planning.^6^

Multiparametric Magnetic Resonance Imaging (mpMRI) has been widely used for detection of PCa in recent years, because of its high sensitivity and negative predictive value for clinically significant PCa.^7^ Typically, mpMRI consists of an anatomical T2‐weigted (T2W) imaging sequence combined with functional diffusion‐ (DWI) and perfusion (DCE) weighted imaging. However, using a reduced biparametric MRI (bpMRI) protocol, including only T2W and DWI (ADC, apparent diffusion coefficient) is increasingly being studied to reduce costs and decrease image acquisition time while preserving accuracy for PCa diagnosis.^8^, ^9^ In clinical settings the interpretation of prostate MRI is based on the clinical guideline *prostate imaging reporting and data system version 2* (PI‐RADS v2).^10^ PI‐RADS v2 uses a dominant MRI sequence based on zonal location for lesion scoring (DWI for peripheral zone (PZ) lesions and T2W for transitional zone (TZ) lesions) since the zones differ significantly in both biological and imaging features.^11^ DCE imaging is used for equivocal findings in PZ but is not used for TZ lesions.^10^

Evidence suggests that mpMRI also has the ability to noninvasively assess the GS and could be used in the treatment planning.^12^, ^13^ As the analysis of prostate mpMRI is time‐consuming, complex and affected by interobserver variability, computer‐aided diagnostic (CAD) systems are increasingly being designed to assist radiologists in their work and could overcome the abovementioned limitations. Building a CAD system to accurately determine the true pretherapeutic GS can potentially help identify patients suitable for different treatment options.^14^

Current CAD systems have been limited to a two‐ or three‐tier classification of PCa lesions.

The two‐tier systems were designed to differentiation between malignant and nonmalignant prostate tissue, or separate indolent/low grade (3 + 3) from clinically significant/high grade (≥3 + 4) disease.^15^, ^16^ Only one study investigated a three‐tier (low, intermediate, and high grade) system and reported low performance compared to their two‐tier system.^12^ Moreover, the majority of studies using CAD systems are further limited to include only one prostatic zone (often the PZ), which is a major drawback as PCa is a multifocal heterogeneous disease that often occurs in other prostatic zones. A state‐of‐the art study assessing PCa GS classification reported accuracies up to 0.93 in differentiating GS 6 from ≥7 and separating 7(3 + 4) from 7(4 + 3) using T2W and ADC image features.^13^ Another recent study presented an automatic method using convolutional neural networks (CNN) combined with handcrafted features (conventional features, like histogram and texture) for differentiating between noncancerous, indolent (≤6), and clinically significant cancers (GS ≥ 7). They achieved significantly better results compared to the state‐of‐the art system based on handcrafted features alone, with a sensitivity of 100% and a specificity of 76.92% separating GS ≤ 6 from GS ≥ 7 tumors.^17^ Both studies included PCa lesions from the whole prostate but were limited to a two‐tier classification.

Future system should include all prostatic zones and more accurate separation of PCa than two or three groups, as the prognosis and therapeutic options differ for each GS grading.^2^ Therefore, the objective of this study was to assess the use of zonal‐specific image features to accurately determine the GS of PCa lesions from the whole prostate gland using bpMRI.

## 2 METHODS

### 2.A Data

Data used in this study were obtained from The Cancer Imaging Archive sponsored by the international society for optics and photonics (SPIE), National Cancer Institute/ National Institutes of Health (NCI/NIH), The American Association of Physicists in Medicine (AAPM), and Radboud University.^18^ The full dataset consisted of 162 PCa patients with MRI examination including T2W (axial and sagittal), Ktrans (computed from DCE), DWI, and ADC images. A total of 182 PCa lesions were split into a training set (112 lesions) and a test set (70 lesions). Data from the training set were used for this study, as the pathological records (reference standard) have not been released for the testing data set at the time of this study.

For the training set, the location (zone, and center coordinates of the lesion) and the pathological‐defined Prognostic Gleason Grade Group (GG), split into GG 1 (GS = 6), GG 2 (GS 3 + 4 = 7), GG 3 (GS 4 + 3 = 7), GG 4 (GS = 8), and GG 5 (GS = 9–10), were provided.^2^ Table 1 summarizes the data used for this study. All lesions were biopsied under MRI guidance in the scanner. According to the PI‐RADS v2 guidelines we use the dominant MRI sequence based on zonal location. Lesions located in the anterior fibromuscular stroma (AFS) were scored similar to lesions in the TZ, and therefore grouped.^19^

**Table 1:** Data overview

Data	Number
Patients	99
Peripheral zone (PZ)	50
GG1	14
GG2	21
GG3	9
GG4	3
GG5	3
Transitional Zone and Anterior	62
Fibromuscular Stroma (TZ + AFS)	
GG1	22
GG2	20
GG3	11
GG4	5
GG5	4
Total	112

Data used for this study, with number of lesions in each Gleason Grade Group for the peripheral zone, and transitional zone and anterior fibromuscular stroma.

### 2.B MRI image acquisition

All images were acquired on two different Siemens 3T MRI scanners, a Magnetom Trio, and a Skyra, without an endorectal coil. All patient examinations included T2W, DWI (3 b‐values: 50,400 and 800), ADC (calculated by the scanner software), and DCE sequence as described in Ref. 20 A single‐shot echo planar imaging sequence was used to acquire the DWI series with slice thickness of 3.6 mm and in plane resolution of 2 mm. The T2W images had in plane resolution of ≈0.5 mm and slice thickness of 3.6 mm and were acquired using a turbo spin echo sequence. Lastly, the DCE sequence was acquired using a 3‐D turbo flash gradient echo sequence with 4 mm slice thickness, 1.5 mm in plane resolution, and 3.5 s temporal resolution.

### 2.C Preprocessing

**Figure 1:**
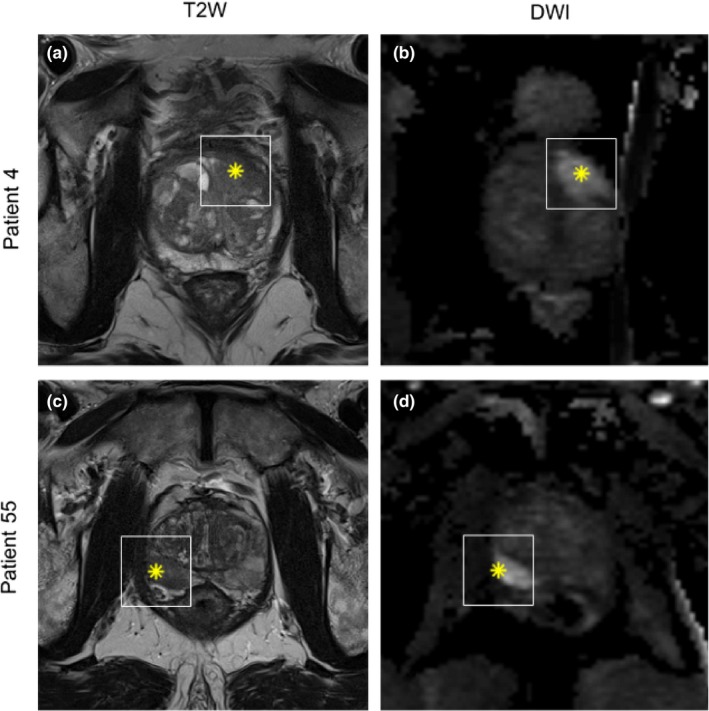
Example of region of interest (white square) around lesion from patient 4 (a and b) with a lesion located in the anterior fibromuscular stroma and patient 55 (c and d) with a lesion in the peripheral zone. Left column is T2W and right column DWI sequence. Asterix inside region of interest denotes the point from where the prostate biopsy was obtained.

All analyses were done using Matlab 2017b. Heavy computations were performed in parallel on a local cluster with 20 workers (processing units) available. Axial T2W and DWI (b‐value = 800) image series were resampled to 0.5 mm × 0.5 mm and T2W series were z‐score normalized to account for interpatient intensity variation. Region of interest (ROI) was defined as a 2D image region of 61 × 61 pixels around the provided lesion coordinate. The size of the ROI was chosen large enough to ensure coverage of largest tumors but as tightly around the lesion as possible. Examples of the ROI around a lesion in AFS and in PZ in shown in Fig. 1. No image coregistration was performed as T2W and DWI series were not used together, and the image fragment of 61 × 61 pixels should ensure that the lesion is within the fragment even with some geometric distortion.

### 2.D Feature extraction

The use of texture features in PCa imaging diagnosis is well demonstrated, even though little is known about the pathophysiology behind.^21^ For this study 11 gray level run length (GLRL) texture statistics derived by Galloway^22^ and 14 Haralick texture features were used.^23^

For all texture features the mean of four directions (0°, 90°, 180°, 270°) with 128 numbers of gray levels were calculated. Histogram‐based metrics such as median, mean, and 10th percentile have previously been shown to correlate with the final GS of PCa lesions.^24^ However, due to substantial overlap in the values and GS, none of these metrics alone can accurately predict the GS. Thirteen histogram features were extracted in this study.

A total of 38 features were extracted from each image fragment, see Table 2.

**Table 2:** Image Features Extracted from DWI and T2W

11 gray level run length texture	14 Haralick texture	13 histogram
1. Short Run Emphasis 2. Long Run Emphasis 3. Gray Level Nonuniformity 4. Run Length Nonuniformity 5. Run Percentage 6. Low Gray Level Run Emphasis 7. High Gray Level Run Emphasis 8. Short Run Low Gray Level Emphasis 9. Short Run High Gray Level Emphasis 10. Long Run Low Gray Level Emphasis 11. Long Run High Gray Level Emphasis	12. Angular Second Moment 13. Contrast 14. Correlation 15. Variance 16. Inverse Difference Moment (Homogeneity) 17. Sum Average 18. Sum Variance 19. Sum Entropy 20. Entropy 21. Difference Variance 22. Difference Entropy 23. Information Measure of Correlation I 24. Information Measure of Correlation II 25. Maximal Correlation Coefficient	26. Mean 27. Variance 28. Skewness 29. Kurtosis 30. Energy 31. Min 32. Max 33. Median 34. 10th percentile 35. 20th percentile 36. 30th percentile 37. 40th percentile 38. 75th percentile

Overview of the 38 features extracted from DWI and T2W from each 61 × 61 pixel image fragment.

### 2.E Feature selection

**Figure 2:**
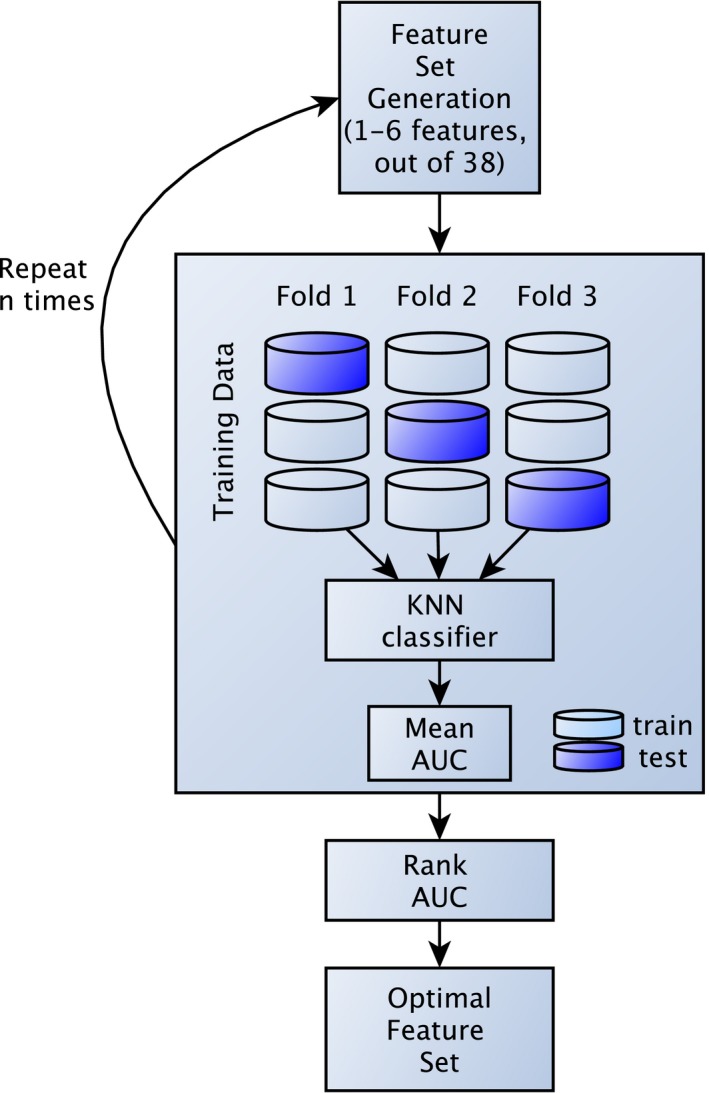
Flowchart of semi‐exhaustive feature selection used in this study. A feature set, with 1–6 features, is generated and evaluated using KNN classifier in a threefold cross validation setup. Mean AUC from the threefolds are ranked to find the most optimal feature set. The process is repeated n times, where n (n = 584.934) equal the number of exhaustive combinations that can be generated out of 38 features (for DWI and T2W), using 1–6 features at the time.

Feature selection is an important task, as removing redundant and irrelevant features can significantly improve the performance of a classifier. Furthermore, a limited number of features decrease the risk of overfitting, especially with small datasets. The optimal feature set is found by exhaustively searching through the whole feature set, however, this task quickly becomes computationally expensive as the number of features increases. For this study, a semi‐exhaustive feature search was performed using all combinations of 1–6 features of the 38 features for each image sequence to find discriminative features without risking overfitting. This resulted in 584.934 feature combinations to be evaluated for each image sequence. A flowchart describing the feature selection is presented in Fig. 2.

### 2.F Classification

K‐Nearest Neighbor (KNN) is a simple nonparametric supervised classifier, which produces a classification output based on a distance search to find the nearest neighbor between training and testing data. KNN was chosen for this study because it is fast, has the ability to learn from small example sets and has shown good results in previous PCa diagnosis mpMRI studies.^16^, ^25^

Each feature combination was evaluated using a KNN classifier with feature normalization and correlation as distance measure.

### 2.G Validation

K‐fold cross‐validation (CV) is a commonly used method to estimate model performance, optimize the use of all samples in small datasets for training and avoiding overfitting. In this study, stratified threefold CV was used. Stratification was used due to imbalance in the dataset in the CV, such that each fold contains approximately the same percentage of “positive” samples as the full data set. This setup will yield three sets of train/test data where all folds will contain samples from both groups even for the smallest groups. Using stratification in CV improves both bias and variance. Mean ROC AUC (Receiver Operator Characteristic area under curve) was calculated from the threefold CV to find the most optimal feature set. This was done for the PZ and TZ + AFS separately. A one‐versus‐rest design was used to construct a binary classifier for each class, using samples from one class as ones, and samples from all other classes as zeros. To classify an unseen sample using one‐versus‐rest design, the sample would be classified using all classifiers and the one yielding the highest probability score would be assigned as class label.

Each prostatic zone (PZ and TZ + AFM) were analyzed using the following binary models:

- GG1 vs rest (GG2‐5)
- GG2 vs rest (GG1 + 3+4 + 5)
- GG1 + 2 vs rest (GG3‐5)
- GG3 vs rest (GG1 + 2+4 + 5)
- GG4 + 5 vs rest (GG1‐3)

Optimally, GG4 and 5 were classified separately, however, due to the low number of samples in these groups, they were merged as one group. As selected patients with GG2 (GS 3 + 4) tumors may be considered suitable for AS,^26^ a model where GG1 and GG2 were grouped was also evaluated.

Evaluation of each binary classification model was performed using AUC in addition to classification accuracy, sensitivity, and specificity.

## 3 RESULTS

At total of 112 lesions were included in this study, with 50 lesions placed in the PZ and 62 in TZ or AFS. Of the 99 included patients (mean age 65 years, range 42–78 years), 87 patients had one cancerous finding (lesion), 11 patients had two findings, and a single patient had three findings.

**Figure 3:**
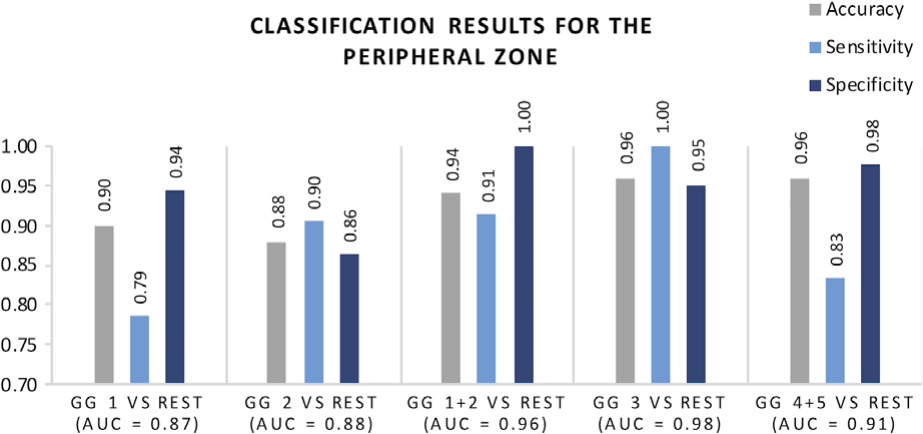
Classification results from threefold cross validation using features extracted from DWI for the peripheral zone (50 lesions). Mean AUC is presented together with accuracy, sensitivity, specificity.

The evaluation results for lesions in the PZ can be seen in Fig. 3. For this zone, AUC values ranged from 0.87 to 0.98, with GG3 vs rest showing the best performance with only two misclassified lesions out of 50 and 100% sensitivity.

**Figure 4:**
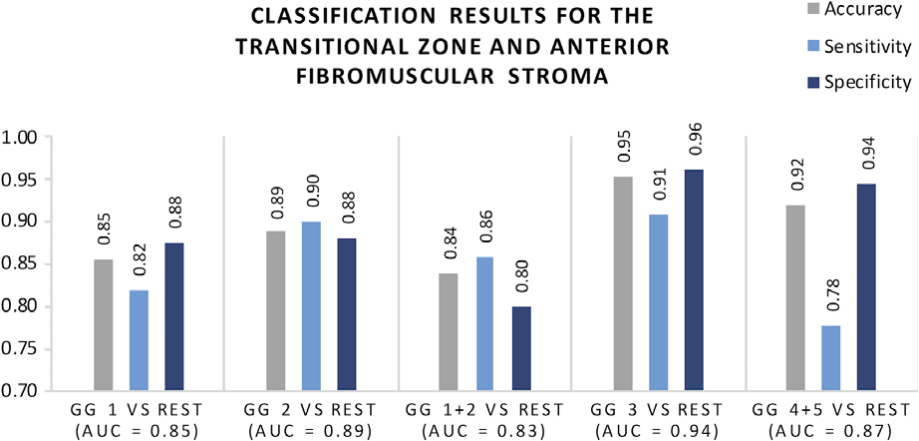
Classification results from threefold cross validation using features extracted from T2W for the transition zone and anterior fibromuscular stroma (62 lesions). Mean AUC is presented together with accuracy, sensitivity, specificity.

The TZ + AFS AUC values ranged between 0.83 and 0.94 as shown in Fig. 4. Similarly, the TZ + AFS GG3 vs rest achieved the best performance with three lesions misclassified, one false negative and two false positives.

For both PZ and TZ + AFS the worst performing classification model was differentiating GG1 from more aggressive lesions.

Overall classification of lesions in the PZ revealed better performance compared to TZ + AFS, with overall mean AUC of 0.92 for PZ and 0.87 for TZ + AFS.

Computational time for feature selection and classification was approx. 6.5 hr for each zone (PZ and TZ + AFS) using 12 of the 20 workers on the local cluster.

Our results indicate that combinations of histogram and texture features achieve the best performance.

The number of features used for the classification models ranged from 5 to 6 for DWI in PZ (see Table 3) and 4 to 6 features for T2W in TZ + AFS (see Table 4). Histogram features were the most used features for classifying lesions in the PZ, with 14 histogram features used for the five models. The use of GLRL texture features were limited in this zone with only five features used. Ten Haralick features were used in PZ with correlation features dominating.

**Table 3:** Features used classification of lesions in the peripheral zone

	GG 1 vs rest	GG 2 vs rest	GG 1 + 2 vs rest	GG 3 vs rest	GG 4 + 5 vs rest
GLRL	9,11	3		1	1
Haralick	14	20, 24	12, 25	13, 14	19, 20, 24
Histogram	27, 28, 29	32, 33, 37	30, 34, 35, 36	27, 34, 36	35

Features used for each classification model for lesions in the peripheral zone. The feature number refers to the list of features in Table 2.

**Table 4:** Features used for classification of lesions in transitional zone and anterior fibromuscular stroma

	GG 1 vs rest	GG 2 vs rest	GG 1 + 2 vs rest	GG 3 vs rest	GG 4 + 5 vs rest
GLRL	3, 5	3, 6	4	6, 7	2, 11
Haralick	15, 22	13	13, 16, 22		14, 19
Histogram	35, 37	35, 38	31, 33	32, 38	31

Features used for each classification model for lesions in transitional zone and anterior fibromuscular stroma. The feature number refers to the list of features in Table 2.

For TZ + AFS, the use of all three feature groups were more equal; nine GLRL texture, eight Haralick texture, and nine histogram features. Only seven of the features were not used in any models in neither PZ nor TZ + AFS.

## 4 DISCUSSION

The aim of this study was to determine the ability of imaging features extracted from bpMRI to accurately determine the pathological Gleason grade of 112 PCa lesions from 99 patients. We found that AUC values using our method are comparable to, or higher than, previously published studies using 2‐tier classification algorithms (i.e., low vs high grade or benign vs malignant).

Interestingly, classifying GG3 revealed the best results for both PZ and TZ + AFS. Only one GG3 was misclassified (false negative) in TZ + AFS and none in the PZ. For GG4 + 5 only one false negative sample was found in PZ. However, since the sensitivity is highly susceptible to one or two false negatives with low number of true positive samples, the sensitivity was as low as 0.83 in PZ. The two false negative GG4 + 5 in TZ + AFS resulted in the lowest sensitivity of 0.78.

For PZ, GG1 + 2 vs higher GG showed good performance with only one false positive and one false negative. For TZ + AFS, however, ten lesions of 62 were misclassified, which is the worst performance presented in this study. This could suggest, that the selected features are specific for each GG and the six features used for this model lack differentiability for grouping GG.

Our results are similar to other studies confirming that mpMRI can be used to classify PCa lesions into grade categories. The winning group for the recent PROSTATEx challenge in differentiating between significant (GS ≥ 6) and nonsignificant (GS < 6) lesion reached an AUC of 0.87 for their model among 33 participating groups.^27^ An AUC of 0.92 was obtained using a KNN‐classifier with textural and statistical features from T2W, DWI, ADC, and Ktrans for differentiation PCa from benign conditions.^16^ Several studies have previously investigated the use of histogram and texture features extracted from mpMRI for PCa imaging.^14^ Both texture and histogram features have also previously been shown to correlate well with PCa aggressiveness.^24^, ^28^ Although texture features have been used for both PCa detection and assessment of aggressiveness, the underlying biology is not yet clear.^21^

Previous works within mpMRI PCa imaging have used different feature selection methods like filter, wrapper, and embedded.^13^, ^29^ For this study we chose to use a semi‐exhaustive features search. The specific choice of selection method is based on the specific application, since an overall aim is to minimize bias, avoid overfitting, and obtain good classification performance. Sequential feature selection methods, like forward and backward selection, were investigated (results not presented), but we found that it quickly got trapped in local minima (e.g., finding one feature, which was descriptive for a particular fold, but not representative for the full dataset alone). Finding local minima is a known disadvantage of sequential selection methods.^30^ Including some randomness into the algorithm might be able to solve the problem but was not investigated in this study.

All models in this study used both histogram, GLRL and Haralick texture features, and the features differed for each GG and zone of the prostate. A recent multi‐institutional study also showed that mpMRI features for PCa detection in PZ differ from those in the TZ.^31^ This fits well with the PIRADS v2 guidelines suggesting that the prostatic zones should be analyzed separately.^10^ Knowledge about zone should be available for automatic system, either from an automatic detection algorithm or from manual detection by a radiologist and can therefore easily be included in assessment models.

Clinical factors, like patient age, PSA (prostate specific antigen), prostate/lesion volume, and T‐stage might improve the performance of the models and could be included in future models. However, one study did include patient characteristics and did not see any improvement in AUC.^16^ According to PIRADS v2 both ADC map and high b‐value images should be included in the PCa analysis. We choose to focus our analysis in the PZ on high b‐value DWI image series. For future studies it might be favorable to use the ADC map or to combine DWI and ADC for GG assessment.

A KNN classifier was used in this study because it is fast and works well with small datasets. We did not consider other classifiers, because KNN performed well for our data. Other popular classifiers include SVM and Naïve Bayes and could be investigated for comparison. Mean AUC was used to find the most optimal features in this study. AUC is a popular metric for evaluating classifier performance and has been proven better than accuracy, both empirically and theoretically. Furthermore, the use of AUC makes it possible to compare the performance of the classifier to those of others, as AUC is a commonly reported metric. However, other metrics, like accuracy or F‐score could also be considered.^32^ The choice of evaluation metrics is application‐dependent and should be based on the classification model and data set; for example, accuracy may yield overoptimistic results for imbalanced class distributions, as algorithms tend to favor the class with most samples.^32^ The metric used might be altered to either value high sensitivity or specificity depending on the clinical situation. For example, when determining if a patient is eligibile for AS it might be favorable to obtain a high specificity for GG1 to make sure that those classified as GG1 with high probability are GG1. Including a patient with high grade PCa into AS could cause undertreatment.

We acknowledge some limitations to the present study; First, it is a limitation that the models were not tested on the test set (70 lesions) from the challenge. This would evaluate the true predictive performance of the models by testing on an independent dataset, which is generally recommended. Second, a future study should include the separation of GG4 and GG5. This was not done in this study, due to the low number of samples in these two groups (3 and 3 for PZ and 5 and 4 for TZ + AFS for GG4 and GG5, respectively). Finally, as no lesion delineation was available for this study, a squared ROI around the lesion center was chosen for feature extraction. As the lesion size varies, it is likely that some noncancerous tissue is included in the ROI and for some lesions not included the whole lesion. Previous studies have shown that delineation of the entire lesion improves accuracy compared to bounding box approach.^33^ A lesion delineation might improve the models; however, such delineation requires experienced personnel and is very time‐consuming. If delineation could significantly improve the models, it should be done automatically in order to minimize the workload.

A substantial amount of papers has been published on automatic PCa detection models.^14^ Combining such a model with an automatic assessment of GG could aid radiologists in their daily work and hopefully improve the pretherapeutic risk assessment of PCa patients. Such a system would need to be validated in clinical settings to determine its performance.

## 5 CONCLUSION

In conclusion, this study showed that zonal‐specific imaging features from DWI and T2W MRI enables automatic differentiate between GG in PCa lesions with promising results. Features used for all the binary classification models included both texture and histogram features.

## CONFLICT OF INTEREST

The authors declare no conflict of interest.
